# Glaucomatous Patterns in Frequency Doubling Technology (FDT) Perimetry Data Identified by Unsupervised Machine Learning Classifiers

**DOI:** 10.1371/journal.pone.0085941

**Published:** 2014-01-30

**Authors:** Christopher Bowd, Robert N. Weinreb, Madhusudhanan Balasubramanian, Intae Lee, Giljin Jang, Siamak Yousefi, Linda M. Zangwill, Felipe A. Medeiros, Christopher A. Girkin, Jeffrey M. Liebmann, Michael H. Goldbaum

**Affiliations:** 1 Hamilton Glaucoma Center, Department of Ophthalmology, University of California, San Diego, La Jolla, California, United States of America; 2 School of Electrical and Computer Engineering, Ulsan National Institute of Science and Technology, Ulsan, South Korea; 3 Department of Ophthalmology, University of Alabama at Birmingham, Birmingham, Alabama, United States of America; 4 Department of Ophthalmology, New York University School of Medicine, New York, New York, United States of America; 5 New York Eye and Ear Infirmary, New York, New York, United States of America; Duke University, United States of America

## Abstract

**Purpose:**

The variational Bayesian independent component analysis-mixture model (VIM), an unsupervised machine-learning classifier, was used to automatically separate Matrix Frequency Doubling Technology (FDT) perimetry data into clusters of healthy and glaucomatous eyes, and to identify axes representing statistically independent patterns of defect in the glaucoma clusters.

**Methods:**

FDT measurements were obtained from 1,190 eyes with normal FDT results and 786 eyes with abnormal FDT results from the UCSD-based Diagnostic Innovations in Glaucoma Study (DIGS) and African Descent and Glaucoma Evaluation Study (ADAGES). For all eyes, VIM input was 52 threshold test points from the 24-2 test pattern, plus age.

**Results:**

FDT mean deviation was −1.00 dB (S.D. = 2.80 dB) and −5.57 dB (S.D. = 5.09 dB) in FDT-normal eyes and FDT-abnormal eyes, respectively (p<0.001). VIM identified meaningful clusters of FDT data and positioned a set of statistically independent axes through the mean of each cluster. The optimal VIM model separated the FDT fields into 3 clusters. Cluster *N* contained primarily normal fields (1109/1190, specificity 93.1%) and clusters *G_1_* and *G_2_* combined, contained primarily abnormal fields (651/786, sensitivity 82.8%). For clusters *G_1_* and *G_2_* the optimal number of axes were 2 and 5, respectively. Patterns automatically generated along axes within the glaucoma clusters were similar to those known to be indicative of glaucoma. Fields located farther from the normal mean on each glaucoma axis showed increasing field defect severity.

**Conclusions:**

VIM successfully separated FDT fields from healthy and glaucoma eyes without *a priori* information about class membership, and identified familiar glaucomatous patterns of loss.

## Introduction

A number of previous studies have used supervised machine-learning techniques to separate healthy from glaucomatous eyes successfully, based on visual function and optical imaging data. [1]–[20] In several instances, machine-learning classifiers (MLCs) have outperformed commercially available software-generated parameters at this task. [6]–[8], [15], [18] Supervised MLCs are trained with labeled examples of class membership (e.g., healthy or glaucoma), preferably based on a teaching label other than the test being assessed. [8] For example the presence of glaucomatous optic neuropathy (GON) can indicate which eyes have glaucoma when assessing visual field-based MLCs, and the presence of visual field defects can indicate which eyes have glaucoma when assessing optical imaging-based MLCs. [21] The MLCs then “learn” to separate healthy and glaucomatous eyes in a training set and the performance (i.e., diagnostic accuracy) of each MLC is assessed on a separate test set not used during training (often using k-fold cross validation, holdout method, or bootstrapping).

An alternate class of MLCs, based on unsupervised learning, also has been employed to identify healthy and glaucomatous eyes, based on visual field data. [22]–[24] Unsupervised learning is a technique that discerns how the data are organized by learning to separate data into statistically independent groups by cluster analysis, or into representative axes by component analysis, without *a priori* information regarding class membership. For instance, component analysis can decompose data by projecting multidimensional data onto *n* axes that meaningfully represent the data.

Independent component analysis (ICA) [25] is an unsupervised classification method that reveals a single set of independent axes underlying sets of random variables. ICA has proven highly successful for noise reduction in a wide range of applications. [26]–[28] However, there are data distributions where components are nonlinearly related or clustered such that they are difficult to describe by a single ICA model, for example, perimetric visual field results from a mixture of healthy and glaucomatous eyes. In these cases, nonlinear mixture model ICA can extend the linear ICA model by learning multiple ICA models and weighting them in a probabilistic (i.e., Bayesian) manner. [25] The ICA mixture model learns the number of clusters and orients statistically independent axes within each cluster. The variational Bayesian framework helps to capture the number of axes in the local axis set and reduces computational complexity. [29] The amalgamation of all these processes is the unsupervised variational Bayesian independent component analysis-mixture model (henceforth, called VIM).

We previously applied VIM to standard automated perimetry (SAP) results from glaucoma patients. Each axis identified by VIM represented a glaucomatous visual field defect pattern, and the severity of that pattern was organized from mild to advanced along each axis. Although identified automatically using mathematical techniques and no human input, VIM for SAP data identified patterns that were similar to those known to be indicative of glaucoma based on decades of expert visual field assessment [24].

Frequency Doubling Technology (FDT) stimuli test the responses of a subset of all available retinal ganglion cells that have different temporal and spatial summation properties compared to those tested using SAP. [30] It is currently undetermined if FDT perimetry data can similarly be organized by VIM into meaningful patterns and axes. The purpose of this study is to determine if VIM can separate a set of normal and glaucomatous FDT fields into acceptable clusters of normal and glaucomatous eyes and to determine if this technique can identify axes representing statistically independent patterns of defect within the glaucoma clusters. If independent axes are identifiable within each glaucoma cluster, future work could use severity changes along the axes composing these clusters to describe glaucomatous progression in FDT data [31].

## Methods

### Study Participants

Individuals included in the current study were participants in the University of California, San Diego (UCSD)-based Diagnostic Innovations in Glaucoma Study (DIGS) and African Descent and Glaucoma Evaluation Study (ADAGES, which also includes participants from University of Alabama, Birmingham, UAB; and New York Eye and Ear Infirmary, NYEE). In total, FDT results from 1,976 eyes of 1,136 individuals were studied.

Each study participant underwent a comprehensive ophthalmologic evaluation including review of medical history, best-corrected visual acuity testing, slit-lamp biomicroscopy, intraocular pressure measurement with Goldmann applanation tonometry, gonioscopy, dilated fundus examination with a 78 diopter lens, simultaneous stereoscopic optic disc photography (TRC-SS, Topcon Instruments Corp. of America, Paramus, NJ), and SAP using the 24-2 SITA Standard test strategy (Humphrey Field Analyzer II, Carl Zeiss Meditec, Dublin, CA). To be included in the study, participants had to have a best-corrected acuity better than or equal to 20/40, spherical refraction within ±5.0 D and cylinder correction within ±3.0 D at baseline, and open angles on gonioscopy. Eyes with non-glaucomatous optic neuropathy, uveitis or coexisting retinal disease that could affect visual fields were excluded.

This research followed the tenets of the Declaration of Helsinki and Health Insurance Portability and Accountability Act guidelines. All study participants provided written informed consent and the UCSD, UAB, and NYEE Human Research Protection Programs approved all methodology.

### Frequency Doubling Technology (FDT) Perimetry Testing

Each participant was tested using FDT with the Humphrey Matrix (24-2 test pattern) FDT Visual Field Instrument (Carl Zeiss Meditec, Dublin, California, USA) with Welch-Allyn technology (Skaneateles Falls, New York, USA) using the Zippy Estimation by Sequential Testing (ZEST) thresholding algorithm. [32], [33] FDT measures the contrast necessary to detect vertical grating targets that undergo counter-phase flicker. Each target subtends 5 degrees of visual angle and has a spatial frequency of 0.5 cycle/degree and counter phases with a temporal frequency of 18 Hz. The test is based on the frequency-doubling illusion and is a sensitive way to measure glaucomatous visual field loss. In addition, the variability of FDT Matrix measurements is less affected by disease-related decreases in sensitivity than the variability of SAP measurements. [34] The details of this test have been described elsewhere [35].

For the purpose of assessing the specificity and sensitivity of VIM-defined clusters, FDT Matrix results from each study eye were classified as within normal limits (i.e., healthy based on FDT results, 1,190 eyes) or abnormal [786 eyes with FDT Glaucoma Hemifield Test (GHT) outside of normal limits or Pattern Standard Deviation ≤5%], based on the instrument’s normative database. All FDT results were reliable, defined as false positives, fixation losses and false negatives ≤33%. Mean reliability results were 2.86%, 5.51% and 1.61% for false positives, fixation losses and false negatives, respectively.

Previous studies using unsupervised classifiers to identify patterns of visual field defect in glaucoma eyes used glaucomatous optic neuropathy (GON), as determined by stereophotograph assessment, as an indicator of disease. [23], [24] Because some eyes with GON have normal appearing visual fields and some eyes with abnormal visual fields do not have glaucomatous optic neuropathy, and since the goal of this study was to understand the structure of the data rather than diagnosis, in particular to find axes that represented visual field patterns within the data, we considered that the truth values used to validate the clusters that best separated glaucoma and normal eyes should be based on FTD visual field results instead of GON. We hypothesized that clusters that best separated healthy and glaucoma results would lead to the axes within the clusters that best represented the visual field patterns within the clusters.

FDT mean deviation was −1.00 dB (S.D. = 2.80 dB) in FDT-normal eyes and −5.57 dB (S.D. = 5.09 dB) in FDT-abnormal eyes, respectively (one-tailed t-test, p<0.001). Individuals providing abnormal FDT results in at least one eye were slightly, but significantly, older than individuals providing normal FDT results from both eyes (55.9 years, S.D. = 15.3 years versus 50.0 years, S.D. = 14.7 years, respectively, two-tailed t-test p<0.001).

### Variational Bayesian Independent Components Analysis Mixture Model (VIM) Description

This technique has been described in varying degrees of detail previously, by our collaborators and by us. [22]–[24], [36], [37] As described above, VIM is an amalgamation of multiple ICA models weighted in a probabilistic manner. This combination allows the unsupervised identification of independent clusters of data, each containing statistically independent axes of information. In the current study, VIM training was based on the absolute sensitivity values from each of the 52 visual field test points (excluding blind-spot points) and age (a total of 53 dimensions) from all FDT tests (1,976 in total).

**Figure 1:**
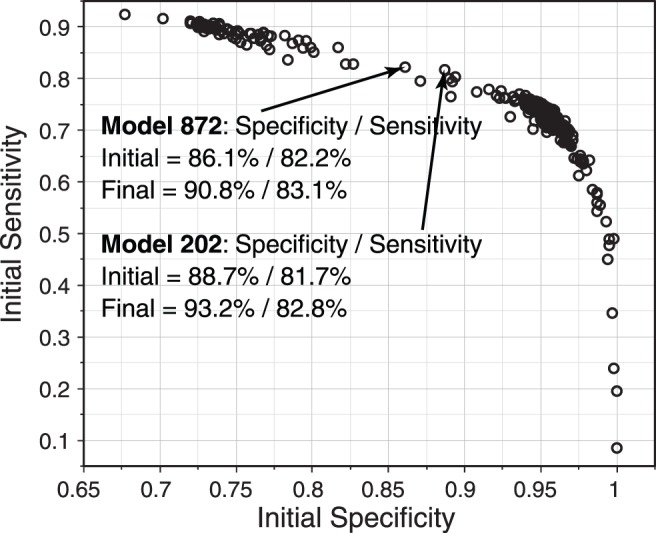
Scatter plot showing sensitivity (Y) and specificity (X) of each of 720 variational Bayesian independent component analysis mixture (VIM) models created from FDT Matrix threshold sensitivities (52 inputs, plus age). Results for the two best models (defined subjectively with a goal of 0.90 specificity and a maximum sensitivity) are shown.

First, several candidate VIM models were created using two to five possible Gaussian clusters, with a maximum of 10 or 20 possible axes per cluster and three, six or eight possible mixture components. These candidate VIM models were repeated with 30 random initializations to avoid finding a locally optimal solution. Thus, a total of 720 VIM models were created (4 trial clusters, 2 axis choices per cluster, 3 mixture component sets, and 30 random initializations), and each model was subjected to 500 iterations of training (a sufficiently high number of iterations, arbitrarily specified, to identify model convergence) in an attempt to identify the models that provided the highest specificity versus sensitivity trade-off (defined with a specificity goal of 0.90). Figure 1 shows the initial specificity and sensitivity (prior to 500 iterations of retraining) of all 720 models tested, and shows the two “best” models.

**Figure 2:**
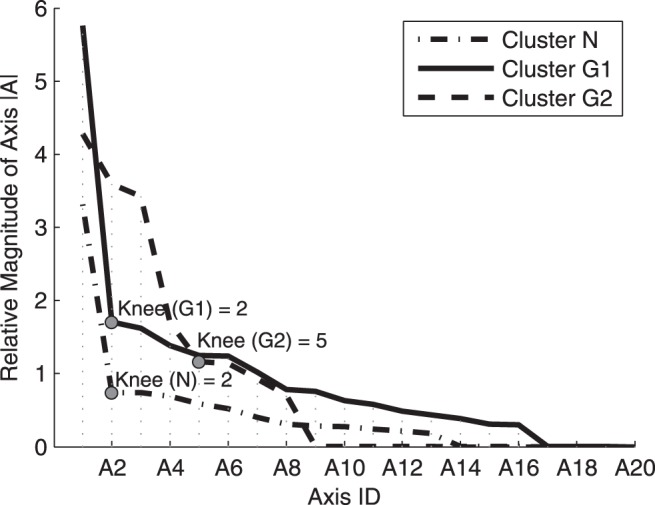
Plot of axis contribution of variational Bayesian independent component analysis mixture (VIM) (Y) versus number of axes (X). Axes beyond the knee point were removed, leaving 2 axes each for Clusters 1 and 2 and 5 axes for Cluster 3. The best VIM model was retrained 500 times, constrained to the reduced number of axes.

The single best model (#202 with the highest specificity versus sensitivity trade-off) maximized specificity and sensitivity using three clusters, each including a maximum of 20 axes. The optimal number of axes within each cluster was manually chosen based on the previously described process of finding the “knee” points. [38] Knee points were chosen by ranking the axes in each cluster based on their length/magnitudes and including the number of axes with the largest relative magnitudes and excluding axes with less significant magnitudes. Figure 2 shows the axes manually selected for each cluster.

The optimal (i.e., best) version of model #202 contained three clusters, C1, C2 and C3, composed of two, two and five axes, respectively. This version was retrained 500 times to determine the final best specificity and sensitivity of the model.

## Results

The optimal VIM model (#202) had an initial specificity of 0.887 and sensitivity of 0.817 (Figure 1). After 500 iterations of retraining, the specificity and sensitivity improved to 0.931 and sensitivity of 0.828, respectively.

The three clusters identified by the best VIM model were one cluster composed mainly of normal FDT fields and two independent clusters composed mainly of abnormal (i.e., glaucomatous) fields. The primarily normal cluster (called cluster *N*) was composed of 1,109 normal fields (89%) and 135 abnormal fields (11%). The first “glaucoma” cluster (called cluster *G1*) was composed of 474 abnormal fields (85%) and 81 normal fields (15%) and the second glaucoma cluster (*G2*) was composed of 177 abnormal fields and 0 normal fields. Table 1 shows the number of normal and abnormal FDT fields assigned to each cluster.

**Table 1:** Number of normal and abnormal FDT fields assigned to each VIM-identified cluster. Overall specificity was 0.931 and sensitivity was 0.828.

FDT field results	Cluster N	Cluster G1	Cluster G2
Normal (n = 1,190)	1,109 (89%)	81 (15%)	0 (0%)
Abnormal (n = 786)	139 (11%)	474 (85%)	177 (100%)
Total (n = 1,976)	1,244	555	177

FDT = Frequency Doubling Technology perimetry, VIM = variational Bayesian independent component analysis mixture model.

Recall that cluster *N* was represented by two axes; cluster *G1* was represented by two axes and cluster *G2* was represented by five axes. VIM projects visual fields along each axis, and the location of the projection on the axis indicates disease severity. The further away in the positive direction a field projection is from the cluster mean, the more severe the visual field defect. Increased distance in the negative direction represents a defect less severe than the mean defect, along a given axis.

**Figure 3:**
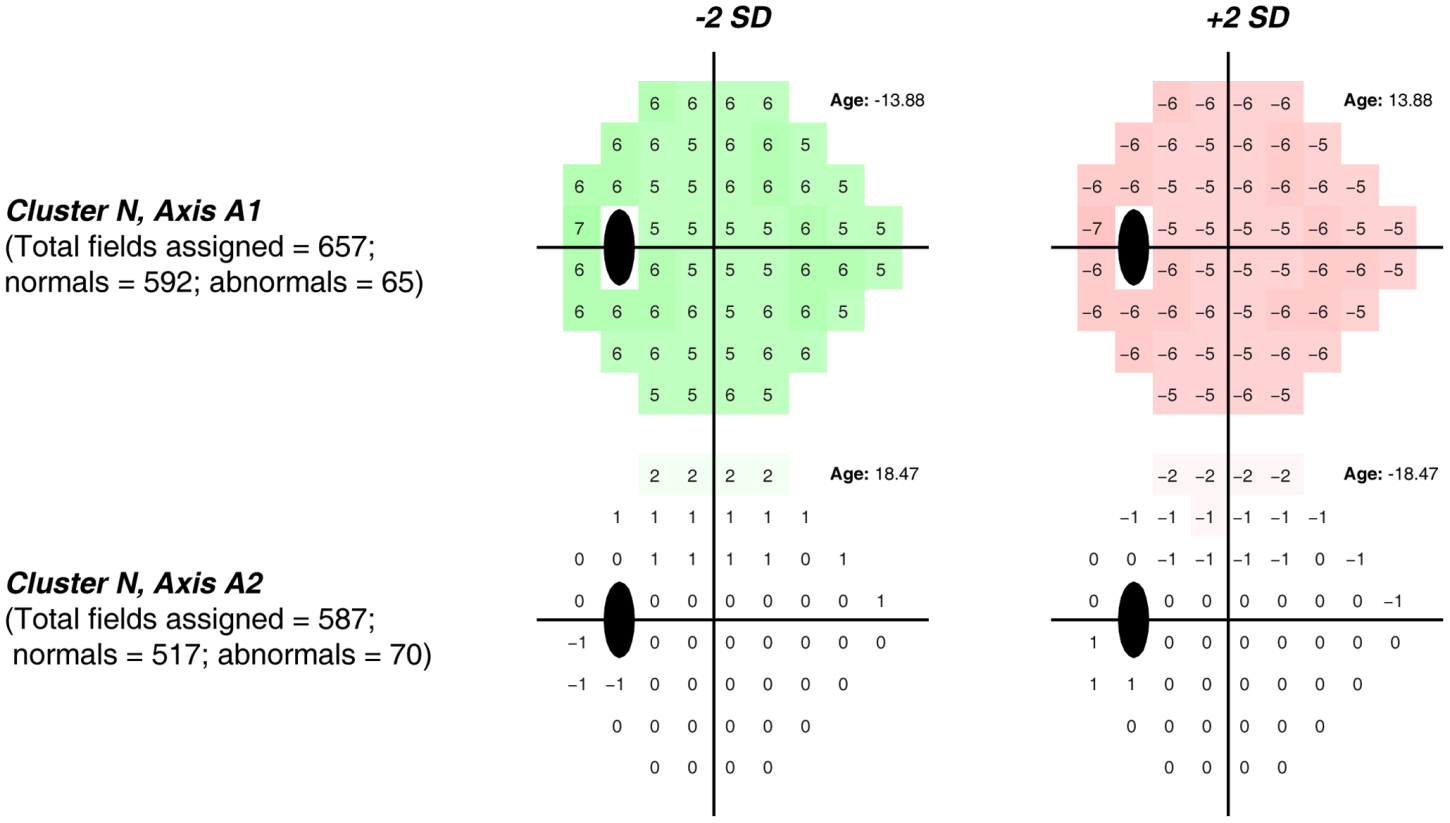
Color-coded displays simulating total deviation plots along with age at −2 and +2 standard deviations of each axis from the centroid of Cluster *N*, that was composed primarily of normal FDT fields. *Axis 1* and *Axis 2* appear normal or near normal. Numerical values shown are simulated total deviation values at each corresponding test point.

Figure 3 shows generated FDT fields and age on VIM axes ±2 standard deviations from the cluster mean for each of the two axes that compose cluster *N*. The color scale simulates a total deviation plot (FDT defined total deviation at each test point with red indicating decreases in sensitivity and green indicating increases in sensitivity, relative to the 53-dimensional normal cluster mean), with the values also expressed for each visual field location. Predictably, both of them appear as normal, or close to normal, fields because 89% of the actual FDT fields clustered there are normal, and the 11% of fields from glaucoma eyes differed little in appearance to the fields from normal eyes. The actual fields closest to *Axis 1* form a set composed of 657 fields, 592 of which are normal and 65 of which are abnormal. The fields closest to *Axis 2* form a set composed of 587 fields, 517 of which are normal and 70 of which are abnormal.

**Figure 4:**
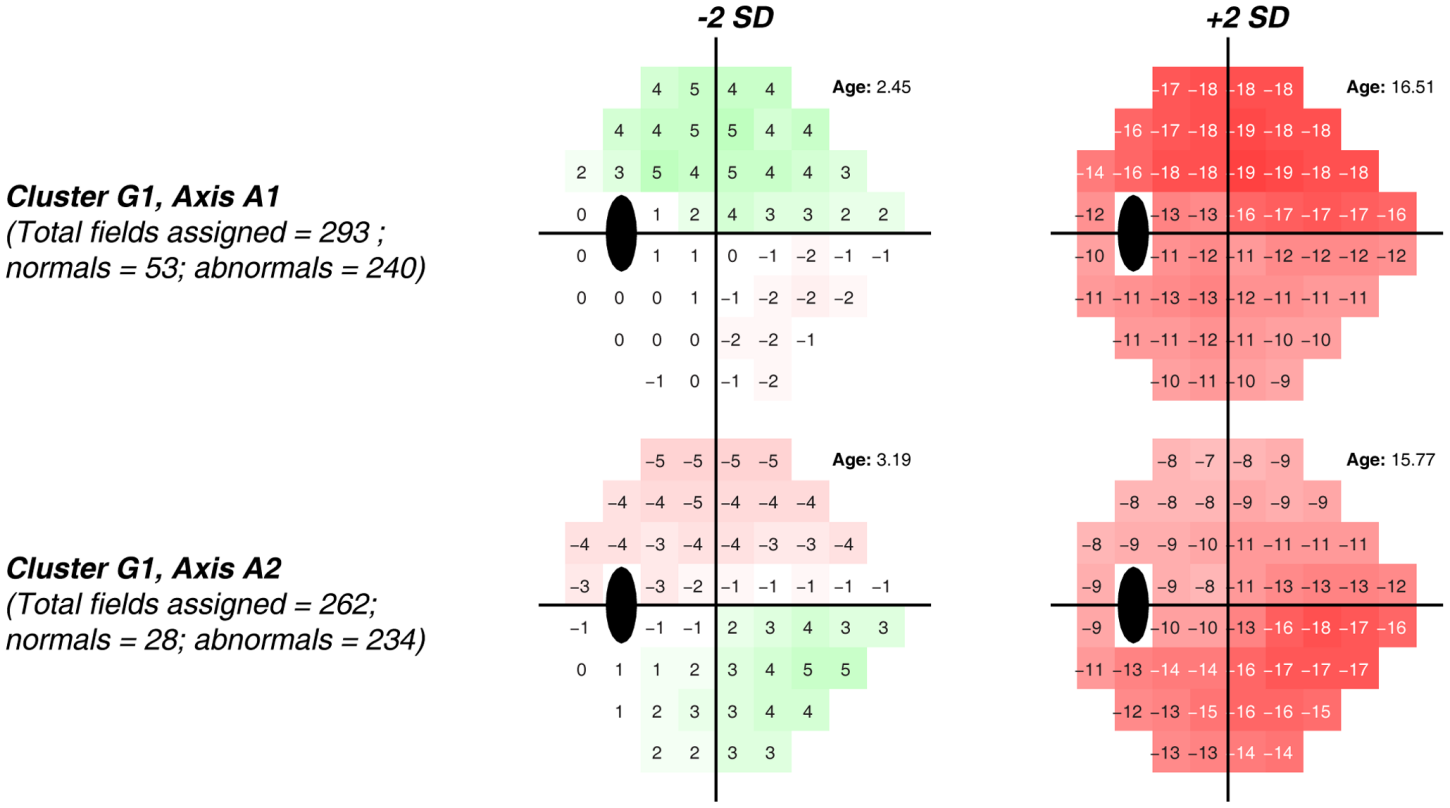
Color-coded displays simulating total deviation plots along with age at −2 and +2 standard deviations of each axis from the centroid of the normal Cluster *N*, that was composed primarily of abnormal FDT fields. *Axis 1* appears to represent primarily moderate superior hemifield defects and *Axis 2* appears to represent primarily moderate inferior hemifield defects (i.e., both are altitudinal defects), both showing less severe, diffuse loss in the opposing hemifield.

Figure 4 shows generated FDT fields and age on VIM axes ±2 standard deviations from the mean of the normal cluster N for each of the two axes that represent cluster *G1*. Regarding visual field patterns generated at +2 standard deviations from the cluster means (i.e., significant, moderate defects), *Axis 1* appears to represent primarily moderate superior hemifield defects and *Axis 2* appears to represent primarily moderate inferior hemifield defects (i.e., both are altitudinal defects), both with diffuse loss in the opposing hemifield. The actual fields closest to *Axis 1* form a set composed of 293 fields, 240 of which are abnormal and 53 of which are normal. The set for *Axis 2* is composed of 262 fields, 234 of which are abnormal and 28 of which are normal.

**Figure 5:**
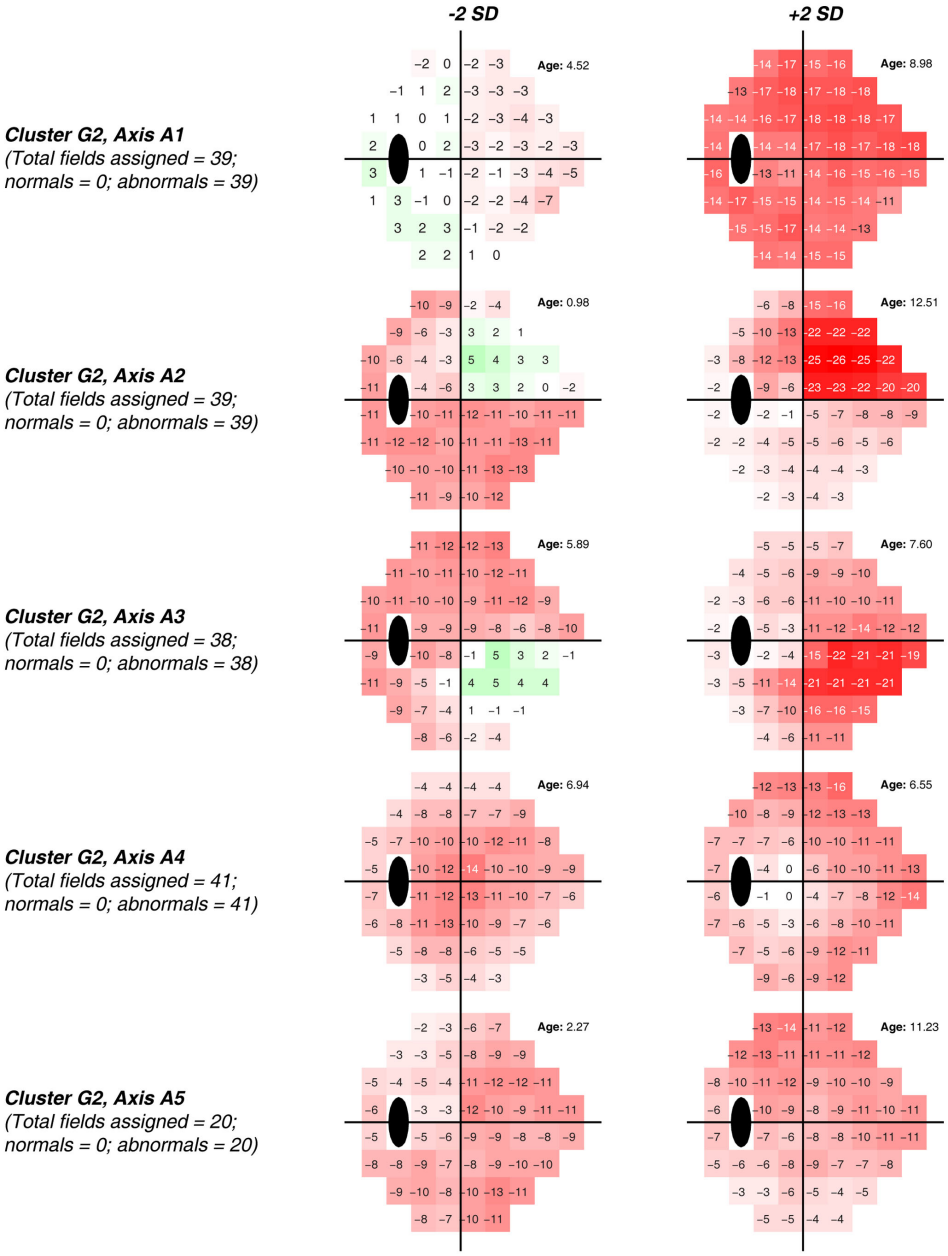
Color-coded displays simulating total deviation plots along with age at −2 and +2 standard deviations of each axis from the centroid of normal Cluster *N*, that was composed entirely of abnormal FDT fields. *Axis 1* appears to represent diffuse moderate visual field loss. *Axis 2* and *Axis 3* appear to represent more severe superior nasal and inferior nasal defects, respectively. *Axis 4* appears to represent combined superior and inferior nasal step defects, and *Axis5* appears to represent a diffuse pattern of loss, primarily localized superiorly.

Figure 5 shows generated FDT fields and age on VIM axes ±2 standard deviations from the mean of the normal cluster N for each of the five axes that represent cluster *G2*. Regarding visual fields placed +2 standard deviations from the cluster means, *Axis 1* appears to represent diffuse moderate visual field loss and the fields closest to this axis form a set composed of 39 fields, all of which are abnormal. *Axis 2* and *Axis 3* appear to represent more severe superior nasal and inferior nasal defects, respectively. The set of fields closest to *Axis 2* is composed of 39 fields, and the set of fields closest *Axis 3* is composed of 38 fields (all abnormal). *Axis 4* (41 fields, all abnormal) has the pattern of an “arrowhead” shaped defect, similar to the pattern observed using the VIM technique to identify patterns of visual field defect in SAP data. [38] Likely, this defect pattern represents combined superior and inferior nasal step defects. Finally, *Axis 5* within this cluster (20 fields, all abnormal) appears to represent a diffuse pattern of loss, primarily localized superiorly.

## Discussion

The variational Bayesian independent component analysis mixture model employed in this study to identify clusters of FDT Matrix visual fields discriminated between normal and abnormal fields with high specificity and sensitivity, without any *a priori* information regarding class membership. We believe this is important because mathematical techniques were used to define clusters with no human input (i.e., FDT fields were not sorted by a trained perimetry expert). In addition, patterns of FDT visual field loss generated on the VIM axes were similar to the patterns uncovered by VIM with SAP reported in previous studies [23], [24], [38], and the visual fields closest to the individual axes were similar for both FDT and SAP data. To allow this comparison, eyes used in the current study are a subset of eyes with FDT exams within six months of the SAP exams used in a previous study.

As an unsupervised learning classifier, VIM relies on a probabilistic measure of the likelihood of class membership to identify normal and abnormal clusters instead of relying on labeling of normal and abnormal visual field “templates” against which classifiers are trained by supervised learning and later tested. Although VIM was not designed specifically to segregate normal and abnormal visual fields, the FDT clusters created by VIM from the structure of the data yielded clusters that classified normal and glaucoma fields with specificity and sensitivity similar to that accomplished by supervised machine learning techniques, using data labeled with the class identity. [7], [8], [11], [15].

Eleven percent of eyes (n = 135) assigned to the VIM normal field (i.e., cluster *N*) were abnormal based on our criteria (they had GHT outside of normal limits or PSD p≤5%, of these 89% were abnormal based on GHT alone). Upon inspection of Matrix printouts, most of these eyes had mild to moderate scattered decreases in thresholds across the visual field with no apparent pattern of defect (i.e., they appeared somewhat noisy), so they likely did not fit into a specific, identifiable glaucoma cluster. They were, however, outside normal limits when compared to the FDT normative database. Conversely, 15% of the eyes (n = 80) in glaucoma cluster *G_1_* were within normal limits based on the FDT normative database. Upon inspection of Matrix printouts, many eyes had mild, scattered decreases in thresholds, although a hypothetical reason for misclassification was not obvious. These results are not wholly unexpected, because the VIM technique described herein does not rely on results from the FDT normative database to identify patterns of defect.

The identifiable patterns of defect (i.e., axes) within each glaucoma cluster generally resembled those that have been shown to be indicative of glaucoma in SAP tests, based on many years of expert assessment. [39]–[43] The optimal number of clusters obtained by *post hoc* analysis of the VIM applied to FDT was three, as it was for SAP [38]; the members of the clusters were similar for FDT and for SAP, the number of axes in each cluster was the same for FDT and SAP [38], and the visual field patterns represented by the axes were similar for FDT and for SAP. The latter observation may not be surprising because recent evidence suggests that the assumed target cells for FDT testing (magnocellular ganglion cells) are sensitive to both FDT-like stimuli and SAP-like stimuli [44] (although see [45]). For both FDT and for SAP, the visual field patterns uncovered by VIM resembled those discovered by human perimetry experts over decades of experience (as previously mentioned). For instance, VIM identified diffuse and altitudinal defects of different severities, in addition to nasal step-like defects (e.g., *G_2_*, *Axis 4*) within the glaucoma clusters.

Age is a significant risk factor of glaucoma and its progression. To study how the age of study eyes influenced the defect patterns identified and generated by the VIM algorithm, we ran the VIM algorithm with the age parameter input set to zero for all study eyes. The VIM model generated without age provided a similar diagnostic accuracy and defect patterns. Therefore, it was evident that age did not significantly affect or bias how the FDT defect patterns were identified and automatically generated by the VIM algorithm.

Although the specificity and sensitivity of VIM-discovered clusters were very good relative to the result of the FDT test (within normal limits versus abnormal), cluster assignment did not always agree with characteristics of the optic nerve and retinal nerve fiber layer, as assessed by masked stereoscopic photograph assessment. This is not unexpected, because several studies have shown a disagreement between classification by visual function and structural assessments (e.g., [46]). This disagreement likely is attributable, in part, to the sensitivity of the tests used and the variability in results, particularly for masked stereophotograph assessment, which is a subjective process. The variability in appearance of healthy optic discs makes this task difficult even with significant training, particularly in the cases of borderline (i.e., suspected glaucoma) discs. A *post-hoc* examination of agreement between VIM-defined clusters and the presence of glaucomatous optic neuropathy (GON, defined based on masked assessment by two independent graders, adjudicated by a third if agreement was not observed) showed that in cluster *N* (the “normal cluster”, n = 1,244; Table 1), 67 (5%) eyes had apparent GON and 136 (11%) eyes had FDT results outside normal limits. Both methods classified as abnormal 10 of the same eyes in this cluster; suggesting misclassification by either method likely was due to the presence of early disease (because agreement between methods usually is better in advanced disease, when defects using both methods are expected). Within the “glaucoma” clusters (*G_1_* and *G_2_*), 278 (38%) eyes had apparent GON and 649 (89%) had abnormal FDT results.

In previous studies [22], [23], [38], VIM applied to SAP created an environment for the development of machine learning methods for detecting progression of glaucomatous field defect. [31] Since the outcome of VIM applied to FDT data was similar to that of SAP, the expectation is that the outcome of VIM analysis of FDT data likewise will be a good environment for analyzing glaucomatous progression.

In summary, VIM was applicable to FDT data. The outcome of the VIM process with FDT data was similar to the outcome with SAP data. Even without foreknowledge of the diagnosis of normal or glaucoma, unsupervised learning analyzing the internal structure of the data yielded separation of normal and glaucomatous eyes as well as can be achieved with supervised learning with foreknowledge of the diagnosis. Variational Bayesian independent component analysis mixture model can find statistically different visual field patterns similar to those identified by human experts. The axis representation of the internal structure of the data arranges mild to severe orientation of each pattern of visual field defect, thus permitting analysis for progression of disease. VIM, both for FDT and SAP, likely provides a good environment for the development of machine learning methods for detecting progression.
